# Discovery of γ-Mangostin as an Amyloidogenesis Inhibitor

**DOI:** 10.1038/srep13570

**Published:** 2015-08-27

**Authors:** Takeshi Yokoyama, Mitsuharu Ueda, Yukio Ando, Mineyuki Mizuguchi

**Affiliations:** 1 Faculty of Pharmaceutical Sciences, University of Toyama, 2630 Sugitani, Toyama 930-0914, Japan; 2 Department of Neurology, Graduate School of Medical Sciences, Kumamoto University, 1-1-1 Honjo, Kumamoto 860-8556, Japan

## Abstract

Transthyretin (TTR) is a homotetrameric protein involved in human hereditary amyloidoses. The discovery and development of small molecules that inhibit the amyloid fibril formation of TTR is one of the therapeutic strategies for these diseases. Herein, we discovered that γ-mangostin (γ-M) is an effective inhibitor against the amyloid fibril formation of V30M amyloidogenic TTR. *In-vitro* binding assays revealed that γ-M was the most potent of the selected xanthone derivatives, and it bound to the thyroxine (T4)-binding sites and stabilized the TTR tetramer. X-ray crystallographic analysis revealed the diagonal binding mode of γ-M and the two binding sites of chloride ions at the T4-binding site. One of the chloride ions was replaced with a water molecule in the α-mangostin complex, which is a methylated derivative of γ-M. The stronger inhibitory potency of γ-M could be explained by the additional hydrogen bonds with the chloride ion. The present study establishes γ-M as a novel inhibitor of TTR fibrillization.

Transthyretin (TTR) is a transporter protein that binds to retinol-binding protein and thyroxine (T4) in human plasma. TTR is also known to be involved in human amyloidosis. Point mutations, such as V30M and L55P, are known to cause familial amyloidotic polyneuropathy (FAP) and familial amyloid cardiomyopathy (FAC)^1^,^2^. Since several physical experiments have indicated that the amyloid fibrils of TTR are formed through denaturation and misfolding, the development of a chemical agent that increases the molecular stability of TTR has been considered an efficient strategy for suppressing TTR amyloidosis^3^,^4^,^5^,^6^. TTR is a homotetrameric protein and has two funnel-shaped T4-binding sites that are located at the dimer-dimer interface. Several small molecules bind to the T4-binding sites, stabilize the TTR tetramer and inhibit TTR amyloid fibril formation. Several natural products, including flavonoids, caffeic acid phenethyl ester, curcumin, nordihydroguaiaretic acid and resveratrol, inhibit TTR amyloid fibril formation^7^,^8^,^9^,^10^,^11^. Whereas the typical TTR stabilizers possess two aromatic substructures and linkers^12^, some compounds with a tricyclic ring system have also been identified as TTR stabilizers^11^,^13^,^14^. Some xanthene and xanthone derivatives have also been shown to interact with TTR^13^,^15^. However, in terms of the xanthone derivatives, the molecular interactions and the role of functional groups have not been adequately investigated.

*Garcinia mangostana L.*, commonly known as mangosteen, is a tree that is mainly found in India, Myanmar, Sri Lanka, and Thailand. The pericarp of mangosteen has been used in Thailand as an indigenous medicine for the treatment of skin infections, wounds, and diarrhea. The major bioactive compounds in mangosteen are xanthone derivatives, with α-mangostin (**1**, α-M) and γ-mangostin (**4**, γ-M) being the main derivatives isolated (Fig. 1)^16^,^17^. α-M and γ-M possess not only antioxidant activity but also a wide range of biological activities such as tumor cell-selective apoptosis, and the attenuation of amyloid-β neurotoxicity and anti-inflammation^17^,^18^,^19^,^20^. Although several pharmacological activities of mangostins have been investigated, the potency of these compounds against TTR amyloid fibril formation has not yet been determined. The structure-activity relationships have not been fully explored and the crystal structure of TTR complexed with xanthone has not been solved yet.

In this work, in order to explore the potential of the xanthone derivatives as TTR stabilizers, we tested 12 xanthone derivatives, including α-M and γ-M, by acid-mediated aggregation experiment, pull-down assay, ANS competitive assay and X-ray crystallographic analysis. On the basis of the obtained results, we discuss the inhibitory potency of γ-M, its distinctive binding mode and the role of its hydroxyl groups and dimethylallyl groups.

## Results

### Inhibitory activity of xanthone derivatives

**Figure 1:**
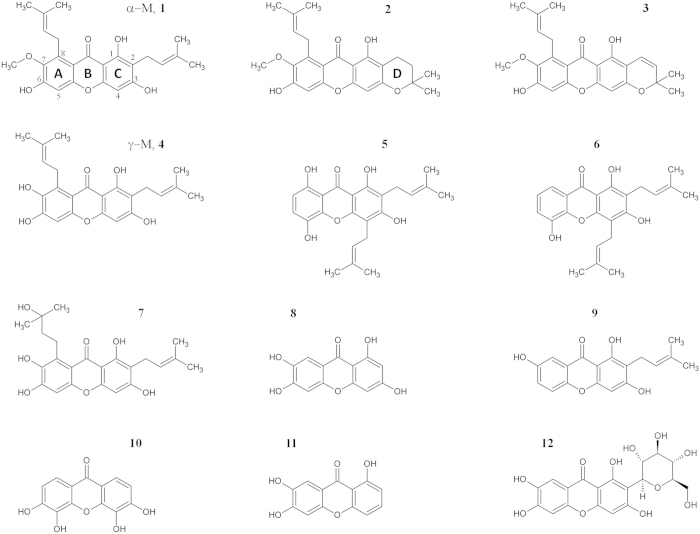
Chemical structures of the selected xanthone derivatives.

V30M mutated TTR (V30M), which is most commonly associated with FAP, was used in this study, along with the following commercially available compounds (Fig. 1 and Table 1): α-mangostin (α-M, **1**), 3-isomangostin (**2**) 9-hydroxycalabaxanthone (**3**), γ-mangostin (γ-M, **4**), gartanin (**5**), 8-deoxygartanin (**6**), garcinone C (**7**), norathyriol (**8**), 1,3,7-trihydroxy-2-prenylxanthone (**9**), 3,4,5,6-tetrahydroxyxanthone (**10**), 1,6,7-trihydroxyxanthone (**11**) and mangiferin (**12**). Amyloid fibril formation of V30M was induced by acidic pH and the amount of fibril was quantified using thioflavin T in the presence of 0–20 μM of the compounds. The EC_50_ values were calculated as previously described^21^. The inhibitory activities against 10 μM V30M are listed in Table 1. The inhibitory potency of γ-M was significantly higher than that of the other tested compounds, with the EC_50_ value being 7.0 μM (Supplementary Fig. S1). The EC_50_ value of α-M was 15 μM, indicating that methylation of the 7-hydroxyl group reduced the inhibitory potency. The inhibitory potency of compound **7**, which is a hydroxyl derivative of γ-M, was lower than that of γ-M but higher than that of α-M. The EC_50_ value of **8**, which has the hydroxyl groups at the same position as γ-M but no dimethylallyl group, was 9.0 μM. Although the inhibitory potency of **8** was significantly lower than that of γ-M (*p* < 0.01), this simple compound could be used as a novel template for an amyloid fibril inhibitor. Compound **2**, which is the structural isomer of α-M, and **3**, which is a desaturated derivative of **2**, had EC_50_ values of >20 μM. The comparison of the inhibition ratio at 20 μM indicated that the inhibitory potencies of **2** and **3** were similar. On the other hand, the EC_50_ of **5**, which is the structural isomer of γ-M, was drastically increased (EC_50_ > 20 μM), and **6**, a dehydroxy derivative of **5**, did not exhibit the inhibitory ability even at a concentration of 20 μM, suggesting that the position of the dimethylallyl groups is important for the inhibitory activity. Although **10** is the structural isomer of **8**, its inhibitory potency was much lower than that of **8**. This result, together with the findings for **11**, suggested that the position and number of hydroxyl groups are important for the inhibitory potency.

### Binding and stabilization by γ-M

**Figure 2:**
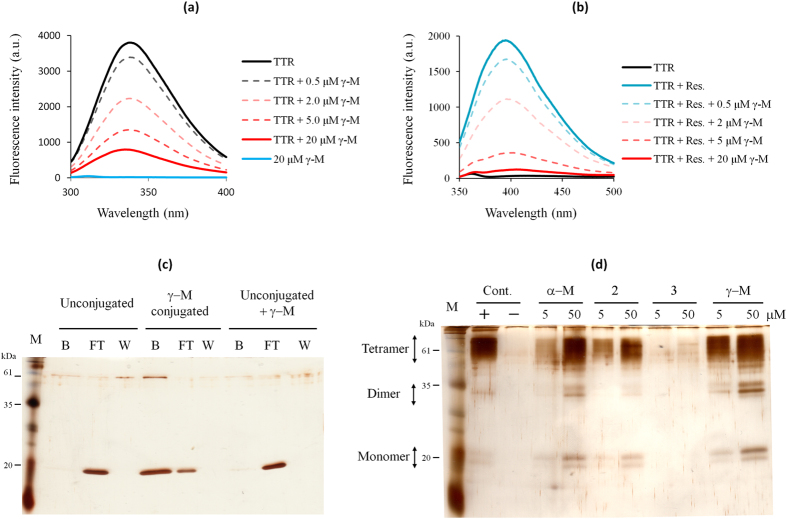
γ-M binds to the T4-binding sites and stabilizes the V30M tetramer. (**a**) Intrinsic fluorescence spectra (300–400 nm) of V30M and upon the addition of γ-M by excitation at 280 nm. (**b**) The fluorescence spectra (350–500 nm) of resveratrol (5 μM) bound to V30M and upon the addition of γ-M by excitation at 320 nm. (**c**) Pull-down assay using CN-Br activated Sepharose. V30M was incubated with γ-M conjugated to Sepharose or Sepharose alone. B indicates the bead fractions, FT indicates the flow-though fractions and W indicates the final wash fractions. V30M was observed in the lane for the γ-M conjugated beads, but not in that for Sepharose alone. (**d**) Effect of γ-M on the acid-mediated quaternary structural changes of TTR (10 μM). The + lane indicates the positive control incubated at pH 8.0 without the compounds. The - lane indicates the negative control incubated at pH 4.5 without the compounds.

In order to determine whether γ-M binds directly to V30M, fluorometric assays were carried out. V30M was titrated with various concentrations of γ-M. A previous study has shown that the binding to TTR induces quenching of the intrinsic fluorescence emission from TTR at 336 nm^22^. In the present study, the addition of γ-M induced similar quenching of the intrinsic fluorescence emission from V30M (Fig. 2a), suggesting the binding of γ-M to V30M. In addition, we performed a competitive binding assay using *trans*-resveratrol, which is known to bind to the T4-binding sites of TTR. The fluorescence emission of resveratrol has been shown to be much lower in the unbound form but to increase upon binding to TTR^23^. In the present study, the fluorescence intensity of resveratrol was significantly decreased by the addition of γ-M (Fig. 2b). This result indicated that γ-M binds to the T4-binding sites of V30M. For additional evidence, a pull-down assay was carried out using Sepharose 4B beads coupled with γ-M. V30M was detected in the Sepharose 4B-γ-M-coupled beads but not in Sepharose 4B beads alone or in Sepharose 4B beads alone with free γ-M (Fig. 2c). This result also indicated that γ-M binds directly to V30M.

The effect of the selected compounds on the quaternary structural stability of V30M at acidic pH was assessed using glutaraldehyde cross-linking followed by SDS-PAGE as previously described^9^,^24^. As shown in Fig. 2d, the tetramer fraction was observed in the presence of α-M and γ-M, and increased in a dose-dependent manner. These results indicated that the mangostins bound to V30M and stabilized the TTR tetramer. Together with the results for the other compounds, these findings suggested that there was a strong correlation between the amount of the tetramer fractions and the inhibition ratio, with a Pearson correlation coefficient of 0.85 (Fig. 2d, Supplementary Fig. S3 and S4). These results indicated that the determination of EC_50_ by thioflavin T assay was not biased by fluorometric interference or the competitive binding of thioflavin T.

### Diagonal binding mode of γ-M

**Figure 3:**
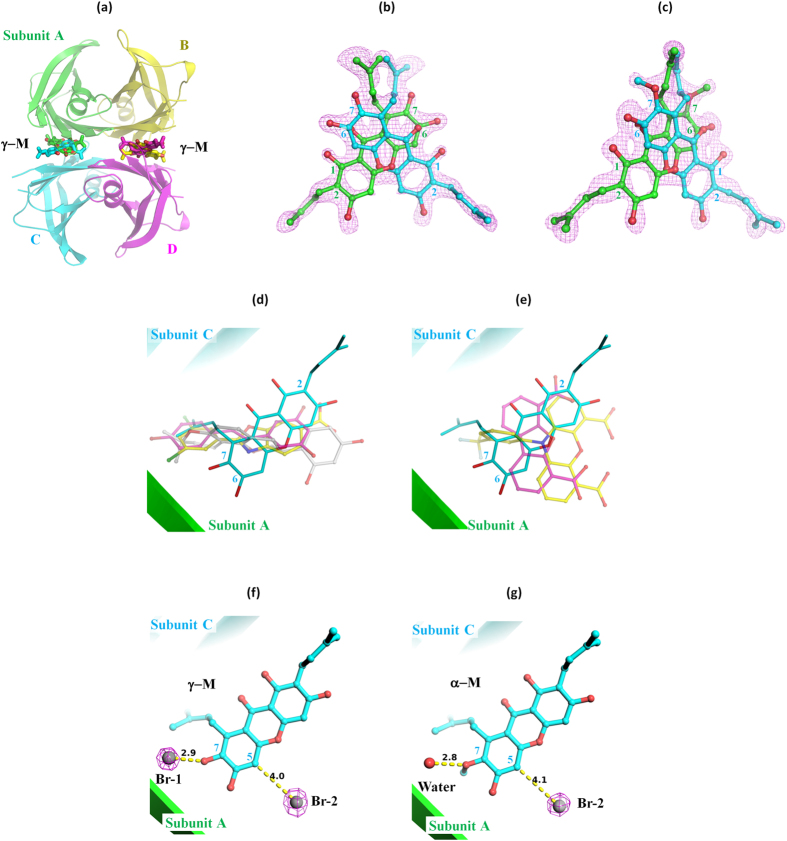
Crystal structures of V30M in complex with γ-M and α-M. (**a**) Overall structure of V30M in complex with γ-M. (**b**) Omit Fourier map of γ-M bound to TTR contoured at 2.8 σ. (**c**) Omit difference Fourier map of α-M bound to TTR contoured at 3.3 σ. (**d**) A comparison of the binding directions with the known stabilizers. The carbon atoms of tafamidis (PDB ID: 3TCT), resveratrol (1DVS) and glabridin (4N87) are colored yellow, magenta and silver, respectively. (**e**) A comparison of the binding directions with the known stabilizers with a tricyclic ring system. The carbon atoms of PHENOX (1DVY) and dibenzofuran-4,6-dicarboxylic acid (1DVU) are colored magenta and yellow, respectively. Anomalous difference Fourier maps of Br derivatives of the γ-M (**f**) and α-M (**g**) complexes contoured at 5.0 σ are also shown.

In order to understand the molecular basis of the inhibitory activity of γ-M, the X-ray crystal structure of V30M in complex with γ-M was determined at 1.5 Å resolution (Supplementary Table S1). To gain additional insight, we also determined the V30M-α-M and V30M-**2** complex structures at 1.4 and 1.9 Å resolution, respectively. All crystals belonged to space group *P*2_1_2_1_2 and were isomorphous, with typical TTR crystal structures deposited in the Protein Data Bank regardless of mutations or ligands. There was a dimer (subunits A and B) in the asymmetric unit, which forms, along with a crystallographically equivalent dimer (subunits C and D), the functional tetramer. Mangostins were found at the T4-binding pockets located between subunits A and C or between subunits B and D on the crystallographic twofold rotation axis as expected from the resveratrol competitive binding assay (Fig. 3a). Since the xanthone derivatives were located on the twofold rotation axis, they were refined as 50% occupancies. Because the difference omit Fourier maps around the 1-hydroxyl, 2-dimethylallyl, 6-hydroxyl, 7-hydroxyl (methoxy for α-M) and dimethylallyl groups were clearly observed, the binding modes could be conclusively determined (Fig. 3b,c). The rmsd value of the xanthone skeletal structure (18 atoms) was 0.36 Å, indicating that the binding positions and directions of γ-M and α-M were almost identical. Only small structural differences were observed when comparing the wild type TTR and V30M crystals in the presence of inhibitors, although the aggregation of V30M is known to be more pronounced than that of the wild type TTR^21^,^25^. It should also be noted that similar binding modes of glabridin have been found in the wild type TTR and V30M complexes. This suggests that the influence of the V30M mutation on the binding geometry would be slight^21^.

Although the mangostins were found at the T4-binding sites, just as in the known TTR stabilizers, the direction of their binding mode was distinctive. The A-ring of γ-M was located at the inner cavity of the T4-binding sites and the C-ring was located at the outer cavity, with the result that 3-OH turned to the solvent. Whereas the typical TTR stabilizers with two aromatic rings and a linker (e.g., tafamidis, resveratrol and glabridin) are known to bind along the twofold axis, the mangostins bound diagonally against the twofold axis (Fig. 3d)^11^,^21^,^26^. Comparison with the other known inhibitors with a tricyclic ring system, such as phenox and dibenzofuran-4,6-dicarboxylic acid, also revealed that the tricyclic rings of mangostins did not fit to those of the inhibitors (Fig. 3e)^11^. The remarkable features of the binding included the 2-dimethylallyl group pointing to the side chain of V121 of one subunit (subunit C in Fig. 3d,e) and the 6- and 7-hydroxyl groups pointing to another subunit (subunit A in Fig. 3d,e). These functional groups would be important for the novel binding mode of the mangostins.

### Chloride ions associated with the binding of γ-M

**Figure 4:**
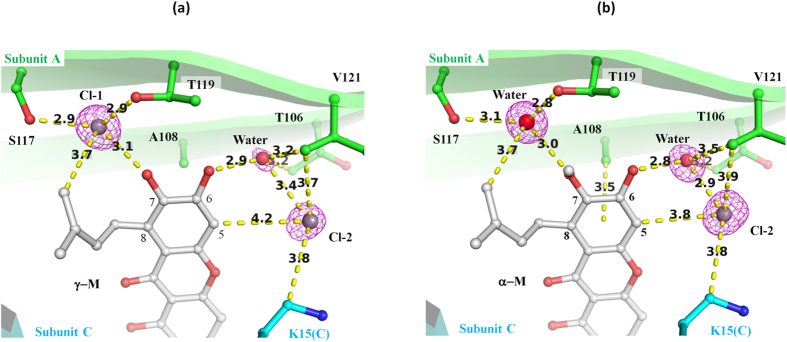
The hydrogen-bond network of chloride ions and water molecules. Atomic interactions can be seen around the A-ring of γ-M (**a**) and α-M (**b**). The omit maps were contoured at 3.3 σ and are shown as a magenta-colored mesh. The carbon atoms of subunits A and C and the ligands are colored in green, cyan and silver, respectively. The oxygen, nitrogen and chloride are colored red, blue and gray, respectively. The hydrogen bonds and CH∙∙∙π interactions are indicated as yellow dashed lines.

Two strong Fourier peaks were observed around S117, T119, 5-H, 7-OH and 8-dimethylallyl of γ-M (Fig. 4a,b) in the T4-binding sites. Since these peaks were previously identified as a halide ion binding site^27^ and the crystallization conditions included 0.2 M CaCl_2_, it is conceivable that the Fourier peak was derived from chloride ions. In order to determine whether chloride ions were the source of the peak, we performed a single-wavelength anomalous dispersion experiment using the bromide derivatives of the γ- and α-M complex crystals. The anomalous difference map showed that two bromide ions bound to the T4-binding sites with γ-M (Fig. 3f). Since the positions of the Br ions were in accordance with the Fourier peaks which were observed in the native V30M-γ-M complex, we concluded that they were chloride ions but not water molecules. Cl-1 (corresponding to Br-1) was hydrogen-bonded with S117-Oγ (2.9 Å), T119-Oγ^1^ (2.9 Å) and γ-M 6-OH (3.1 Å), and these distances are typical of hydrogen bonds with chloride ions (Fig. 4a)^28^. 8-dimethylallyl of γ-M was 3.7 Å distant from Cl-1, indicating a CH∙∙∙Cl^−^ hydrogen bond. Because halide ions are known to be among the strongest hydrogen bond acceptors, these configurations would be reasonable^28^.

In the case of the bromide derivative of the α-M complex, no Fourier peak was observed at the Br-1 position in the anomalous difference map, whereas Br-2 was observed as a strong peak above 7 σ (Fig. 3g). This result indicated that the atom located at position 1 was a water molecule in the native α-M complex, and not a chloride ion (Fig. 4b). Whereas γ-M can donate a hydrogen atom in the hydrogen bond with the chloride ion, α-M cannot donate a hydrogen because of its 6-OCH_3_ group, and thus the chloride ion could not stably bind to position 1. The difference of these interactions could account for the finding that γ-M showed higher inhibitory potency than α-M. Since the compounds with a hydrogen at the 6-position (**5**, **6** and **9**) showed a lowered inhibitory potency, the hydrogen bond with the chloride ion would be important for the stable binding of γ-mangostin.

Cl-2 was located at the outer cavity and CH∙∙∙Cl^−^ was hydrogen-bonded with 5-H of γ-M (4.2 Å), Cγ of V121 (3.2 Å) and C^ε^ of K15(C) (3.8 Å). Cl-2 was also stabilized by forming a hydrogen bond with a water molecule (3.4 Å). This water molecule was involved in a hydrogen bond network that included the hydrogen bond with 6-OH of γ-M (2.9 Å) and the CH∙∙∙O hydrogen bond with Cγ of V121 (3.2 Å). Since the xanthone derivatives with 5-H or 6-OH (compounds **1**–**4**, **7**, **8** and **10**) showed inhibitory activity, the binding of Cl-2 would be helpful for the stable binding of the xanthone derivatives. In addition, a CH∙∙∙π hydrogen bond was found between the A-ring and methyl group of A108 (Fig. 4a,b). Because similar interactions have been observed in the TTR in complex with glabridin^21^, this interaction would be important for the binding of γ-M.

### Role of the dimethylallyl groups

**Figure 5:**
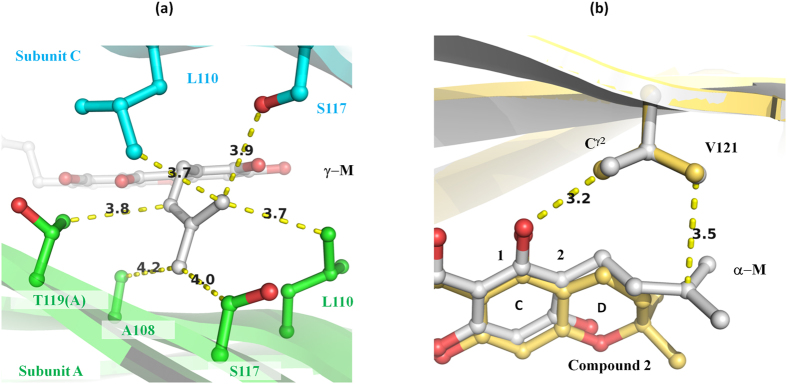
The role of dimethylallyl groups. (**a**) Atomic interactions around the 8-dimethylallyl group of γ-M. All presentations are the same as in Fig. 4. (**b**) Comparison of the interactions of α-M (silver) and compound **2** (light yellow).

In contrast to the other known inhibitors, α- and γ-M possess two distinctive dimethylallyl groups. The 8-dimethylallyl group was sterically surrounded by hydrophobic groups, such as A108, L110, and C^β^ of S117 and C^β^ of T119, and these distances ranged from 3.7 to 4.2 Å in the V30M-γ-M structure (Fig. 5a). The dimethylallyl group would be stabilized by these hydrophobic contacts. Garcinone C (**7**), which is hydroxylated γ-M, showed lower inhibitory potency than γ-M. The 2-methyl-2-butanol group of **7** might not be able to fit into this cavity, since it is larger than the dimethylallyl group. Given the binding mode similar to that of γ-M, it appeared unable to form a hydrogen bond in the **7** complex structure because of its geometry, although S117(C) was located close to the dimethylallyl group. It was suggested that atomic repulsion would have caused lowered inhibitory activity because of the addition of a hydroxyl group.

The results showed that hydrophobic interactions occurred between the 2-dimethylallyl group and the side chain of V121 (Fig. 5b), and a CH∙∙∙O hydrogen bond was formed between the 1-OH and Cγ^2^ of V121. The importance of these interactions could be accounted for by the compound **2** complex structure and the thioflavin T assays. Compounds **2** and **3**, which are cyclized derivatives of α-M, showed significantly lowered inhibitory potencies. The purities of **2** and **3** were 90.8% and 88.8%, respectively, but their impurities could be ignored as long as we compared the inhibition ratio at a 20 μM concentration (EC_50_ values of α-M (61%), **2** (32%) and **3** (29%)). The V30M-**2** structure revealed that the binding mode of **2** was similar to that of α-M and the D-ring; that is, a cyclized dimethylallyl group interacted with the side chain of V121 (Fig. 5b). In the case of α-M, the flexible dimethylallyl group could be adjusted to the shape of the V121 side chain to increase the contact area. In contrast to α-M, the cyclized dimethylallyl of **2** could not fit into V121. The superposed structures clearly showed that α-M interacted with V121 over the whole dimethylallyl group, but only a few atoms were involved in the interaction in the V30M-**2** complex. The lowered inhibitory potency of **2** was likely due to the smaller contact area with V121. These results suggested that the hydrophobic interactions with V121 were important for the binding of mangostins.

In summary, we evaluated the inhibitory activities of the natural polyphenolic xanthones and revealed that γ-mangostin (γ-M, **4**), which is isolated from the pericarp of mangosteen, was the most potent among them, with an EC_50_ value of 7 μM. The fluorometric and pull-down assays indicated that γ-M binds directly to the T4-binding sites of TTR, and the glutaraldehyde cross-linking experiments revealed that γ-M stabilized the TTR tetramer. X-ray crystallographic analysis revealed that γ-M bound to the T4-binding sites in a novel diagonal mode and its binding was associated with two chloride ions. However, one of the chloride ions (CL-1) was replaced with a water molecule in the α-M complex structure. The loss of the hydrogen bonds with chloride ions would explain why γ-M was more potent than α-M. Although γ-M inhibits the TTR amyloidogenesis by stabilizing the TTR tetramer in the same manner as the known stabilizers, its binding to and interaction with TTR show that it belongs to an interesting and novel class of amyloid inhibitors. γ-M may be a good lead compound, or may have potential application as a medicinal agent for TTR amyloidogenesis.

## Methods

### Materials

α-M was purchased from Wako Pure Chemicals (132–16241). γ-M and **9** were purchased from Sigma Aldrich (catalog nos. M6824 and T5955). **2**, **3**, **5** and **6** were purchased from ChromaDex (ASB-00007049, ASB-00004238, ASB-00009310 and ASB-00008369). **7**, **8**, **10** and **11** were purchased from BioBioPha (BBP02049, BBP02007, BBP02022 and BBP02071). **12** was purchased from Indofine Chemical Company (M-001). The purities of most of the compounds were determined as >95% by HPLC, whereas the purities of **2** and **3** were 90.4% and 88.8%, respectively. V30M mutated TTR was prepared as previously described^29^, and its purity was estimated as >90% by SDS-PAGE and Coomassie Brilliant Blue staining.

### Acid-mediated aggregation experiments (thioflavin T assay)

Thioflavin T assay was performed using V30M in [20 mM Tris-HCl pH 8.0 and 150 mM NaCl] and 10 mM stock solutions (ethanol) of xanthone derivatives. Prior to acid-mediated aggregation, 50 μL of V30M (100 μM) was incubated for 30 min in the presence of xanthone derivatives at room temperature. After the incubation, the pH was decreased to 4.5 by adding 450 μL of 50 mM sodium acetate pH 4.5. The sample was gently vortexed and incubated at 310 K for 96 h. At this time, the concentrations of V30M and the xanthone derivatives were 10 μM and 2.5-20 μM, respectively. The solution was 5.5-fold diluted with 200 mM Tris-HCl buffer (pH 8.0). Thioflavin T assays were performed via the addition of 10 mM thioflavin T to 1.8 μM TTR samples. The final concentration of thioflavin T was 20 μM. Fluorescence emission spectra were obtained with excitation and emission wavelengths of 440 and 484 nm, respectively. The fluorescence of the sample without incubation at pH 4.5 was used as a 100% control, and that of the sample in the absence of xanthone derivatives with incubation at pH 4.5 was used as a 0% control. The amyloid fibril inhibitory ratio (%) = 100 × [1 - (fluorescence at each concentration of xanthone derivatives - 100% control)/(0% control - 100% control)]. Amyloid fibril inhibitory curves were fitted using the 4-parameter logistic model by the least squares methods and at least 3 ligand concentrations were used^30^. EC_50_ was estimated according to this model.

### Fluorescence spectroscopy

The binding of γ-M to TTR was confirmed by an intrinsic emission fluorescence spectrum assay and a resveratrol competitive binding assay. For the intrinsic fluorescence, the spectra (300–400 nm) of 2 μM TTR in [50 mM Tris-HCl pH 7.5 and 150 mM NaCl] were recorded by exciting the protein at 280 nm^22^. A γ-M solution was sequentially titrated to the protein solution, and the solutions was stirred and equilibrated for 3 min prior to the measurements. In all cases of the titrations, the maximal dilution was less than 0.3%. The spectra were recorded at 0, 0.5, 2, 5 and 20 μM of γ-M. For the resveratrol competitive binding assay, the spectra (350–500 nm) of 2 μM TTR in [50 mM Tris-HCl pH 7.5 and 150 mM NaCl] were recorded by exciting *trans*-resveratrol at 320 nm^23^. The solutions of resveratrol and γ-M were sequentially added to the protein solution in the same manner as for the intrinsic fluorescence assay.

### Pull-down assay

The pull-down binding assay using CNBr-activated Sepharose 4B (GE Healthcare) was carried out according to previous studies with some modifications^31^,^32^,^33^. γ-M (1 mg) was dissolved in 110 μL of dimethyl sulfoxide (DMSO) and diluted with 390 μL of the coupling buffer [0.1 M NaHCO_3_ pH 8.3, 0.5 M NaCl and 30% DMSO]. Sepharose 4B (25 mg) was resuspended with 0.5 mL of cold 1 mM HCl and washed with the coupling buffer. γ-M in the coupling buffer was added to the Sepharose 4B and incubated at RT for 1.5 h. γ-M conjugated Sepharose 4B was equilibrated with the buffer [0.1 M Tris-HCl pH 8.0 and 0.5 M NaCl] and incubated at RT for 2 h. After washing for 7 cycles with the buffers [0.1 M Tris-HCl pH 8.0 and 0.5 M NaCl] and [0.1 M sodium acetate pH 4.0 and 0.5 M NaCl], γ-M conjugated Sepharose 4B was equilibrated with the binding buffer [50 mM Tris-HCl pH 8, 1 M NaCl, 0.1% Tween 20, 1 mM dithiothreitol, 1 mM ethylenediaminetetraacetic acid pH 8 and 2 μg/mL bovine serum albumin]. V30M was mixed with γ-M conjugated Sepharose 4B and incubated at 4 °C for 12 h. For the control experiment, unconjugated Sepharose 4B was prepared as described above in the absence of γ-M. The beads were washed 5 times with the binding buffer. The proteins bound to the beads were eluted with SDS loading buffer and resolved by SDS-PAGE. The protein bands were visualized with a silver staining kit (Silver Stain Kanto III).

### Glutaraldehyde cross-linking assay

Glutaraldehyde cross-linking experiments were performed as previously described with some minor modifications^9^. The stock solutions of the selected compounds were prepared in ethanol. Prior to the acid-mediated tetramer dissociation, V30M in solution [20 mM Tris-Cl pH 8.0 and 150 mM NaCl] was incubated with the compounds at room temperature for 30 min. After the incubation period, a 2-fold volume of 0.1 M sodium acetate pH 4.5 was added, and then the sample was incubated at 310 K for 10 days. At this stage, the protein concentration was 3.6 μM, and the compound concentrations were 5 and 50 μM. After the incubation period, 50 μL aliquots of the incubated samples were mixed with 2.5 μL of 25% glutaraldehyde and incubated at room temperature for 4 min, and the reactions were terminated by the addition of 5 μL of sodium borohydrate (7% (w/v) in 0.1 N NaOH). The aliquots were then mixed with SDS sample buffer, boiled and resolved by SDS-PAGE. The protein bands were visualized by silver staining.

### Crystallization

10 mM stock solutions of xanthone derivatives were prepared using DMSO. Prior to crystallization, V30M (16.2 mg/mL) was incubated for 30 min in the presence of the xanthone derivatives (final concentration 1–2 mM) containing 30% DMSO. Crystals suitable for X-ray diffraction experiments were obtained by the sitting-drop vapor diffusion method at 293 K. Crystals were grown by mixing 2 μL of TTR-xanthone solution with 2 μL of the crystallization buffer [12–16% polyethylene glycol (PEG) 400, 0.1 M sodium acetate pH 5.5 and 0.2 M CaCl_2_], and the droplets were equilibrated with the reservoir solution [12–16% PEG 400, 0.1 M sodium acetate pH 5.5, 0.2 M CaCl_2_ and 30% DMSO]. For the bromide derivative of V30M-γ-M and α-M, the protein solution was replaced with chloride free buffer [20 mM HEPES pH 7.5 and 200 mM KBr], which was then mixed with 10 mM ligand solution (final concentration: 2 mM (γ-M), 1 mM (α-M)). Crystals were grown by mixing 1.5 μL of the protein solution with 1.5 μL of the crystallization buffer [20% PEG 400, 92 mM MES pH 5.8 and 0.1 M KBr]. Crystals began to appear within one day, and grew for several days. Typical crystal dimensions were 0.2 mm × 0.05 mm × 0.05 mm. Crystals were cryo-protected by 34% PEG 400 and directly frozen in liquid nitrogen until data collection.

### X-ray diffraction data collection, processing and structure refinement

X-ray diffraction data were collected at the beam-lines NW12A and NE3A installed at the Photon Factory Advanced Ring in Japan. Diffraction data sets were processed with HKL2000 and SCALEPACK^34^. The X-ray structure of apo V30M (PDB ID: 4PWE) was used as the initial model^9^. The 3D structures and dictionary data (cif) of xanthone derivatives were obtained from the PRODRG server^35^. The protein structures were refined using PHENIX.REFINE (ver. 1.8–1069) and REFMAC5 (ver. 5.7.0029) with stepwise cycles of manual model building using COOT^36^,^37^,^38^. The final models were evaluated using the Protein Data Bank validation suite^39^. The crystal and refinement data are summarized in Supplementary Table S1, and the experimental conditions of the X-ray diffraction are summarized in Supplementary Table S2. The coordinates and structure factors of V30M-γ-M, V30M-γ-M with bromide ion, V30M-α-M, V30M-α-M with bromide ion and V30M-**2** have been deposited in the Protein Data Bank under the accession codes 4Y9E, 4Y9F, 4Y9B, 4Y9C and 4Y9G, respectively. All structure figures were created using PyMOL^40^.

## Additional Information

**How to cite this article**: Yokoyama, T. *et al.* Discovery of γ-Mangostin as an Amyloidogenesis Inhibitor. *Sci. Rep.* **5**, 13570; doi: 10.1038/srep13570 (2015).

## Supplementary Material

Supplementary Information

## Figures and Tables

**Table 1:** Activities of natural xanthone derivatives.

Compound number	Compound name	EC_50_ (μM)	Inhibition ratio at 20 μM (%)
Control	diflunisal	6.2 ± 0.23*	97 ± 1.7
1	α-mangostin	15 ± 0.75	61 ± 4.7
2	3-isomangostin	>20	32 ± 1.3
3	9-hydroxycalabaxanthone	>20	29 ± 1.5
4	γ-mangostin	7 ± 0.6	97 ± 1.3
5	gartanin	>20	30 ± 0.79
6	8-deoxygartanin	ND	ND
7	garcinone C	10 ± 0.30	83 ± 0.23
8	norathyriol	9.7 ± 0.48	79 ± 0.49
9	1,3,7-trihydroxy-2-prenylxanthone	13 ± 1.1	67 ± 2.7
10	3,4,5,6-tetrahydroxyxanthone	>20	25 ± 0.97
11	1,6,7-trihydroxyxanthone	14 ± 0.51	64 ± 1.2
12	Mangiferin	>20	8.5 ± 2.1

^*^The EC_50_ value was calculated using the fluorescence intensity at 0–40 μM concentration.
