# Supplementary material for: Discovery of γ-Mangostin as an Amyloidogenesis Inhibitor
Source: Sci Rep. 2015 Aug 27;5:13570. doi: 10.1038/srep13570 (PMC4550876; doi:10.1038/srep13570)
Supplement: Supplementary Information [file srep13570-s1.pdf]

SUPPORTING INFORMATION

Discovery of γ-Mangostin as an Amyloidogenesis Inhibitor

Takeshi Yokoyama[a], *Mitsuharu Ueda*[b], *Yukio Ando*[b], *Mineyuki Mizuguchi*[a]*

[a] *Faculty of Pharmaceutical Sciences, University of Toyama, 2630 Sugitani, Toyama 930-0914, Japan*

[b] *Department of Neurology, Graduate School of Medical Sciences, Kumamoto University, 1-1-1 Honjo, Kumamoto 860-8556, Japan*

Corresponding Author

* M. Mizuguchi e-mail: mineyuki@pha.u-toyama.ac.jp;

Supplementary Table S1 and S2 and Supplementary Figures S1, S2, S3, S4 and S5

Supplementary Table S1. Statistics on X-ray data collection and structure refinement

	γ-M	α-M	**2**	γ-M - Br	α-M - Br
**Crystal data**					
Wavelength (Å)	1.0	1.0	1.0	0.91714	0.91714
Resolution range[a] (Å)	33.2–1.5 (1.53–1.5)	27.6–1.4 (1.42–1.40)	30.2–1.9 (1.93–1.90)	28.6–1.5 (1.53–1.5)	28.5–1.5 (1.53–1.5)
Space group	$P2_12_12$	$P2_12_12$	$P2_12_12$	$P2_12_12$	$P2_12_12$
Unit cell (Å)	a=43.4, b=85.8, c=64.1	a=43.6, b=85.4, c=64.7	a=43.0, b=85.2, c=64.5	a=43.4, b=85.7, c=63.4	a=43.1, b=85.6, c=63.6
Observed reflections	230,024	279,805	114,965	333,433	274,834
Unique reflections[a]	38,590 (1,835)	48,115 (2,339)	17,994 (823)	38,151 (1,854)	38,146 (1,812)
R_{merge} (%)[ab]	4.5 (27.4)	6.0 (73.5)	9.0 (61.1)	8.2 (40.3)	7.2 (3.8)
Completeness (%)[a]	97.7 (95.6)	99.3 (98.2)	91.7 (88.2)	99.5 (99.0)	98.3 (95.2)
I/sigma(I)[a]	49.6 (4.3)	26.7 (1.8)	21.0 (1.9)	37.9 (3.6)	24.7 (2.3)
Redundancy[a]	6 (3.8)	5.8 (4.7)	6.3 (3.9)	8.7 (5.8)	7.2 (3.8)
**Refinement data**					
R_{factor}[c] (%)	18.4	19.2	19.6	19.3	20.4
R_{free}[d] (%)	21.9	23.5	24.8	22.1	24.0
R.M.S.D. bonds (Å)	0.014	0.015	0.007	0.016	0.019
R.M.S.D. angles (˚)	1.9	1.8	1.1	1.9	2.1
No. of water and halide	181, 4	239, 2	91, –	176, 8	173, 6
Average B factor (Å^2)					
protein, ligand, halide ion, water	19.1, 22.6, 31.5, 32.8	18.1, 15.9, 31.5, 32.7	19.5, 25.8, –, 23.2	19.1, 16.6, 36.7, 30.6	20.0, 20.2 39.4, 32.7
Ramachandran plot (%)					
Favored, allowed	98, 2	98, 2	96, 4	98, 2	99, 1

[a] Numbers in parentheses refer to the highest resolution shell.

[b] $R_{merge} = \Sigma_{hkl}\Sigma_i|I_i(hkl)\text{-} I_i(\bar{h}\bar{k}\bar{l})|/\Sigma_{hkl}\Sigma_i I_i(hkl)$

[c] $R_{factor} = \Sigma|F_o|\text{-}|F_c|/|F_o|$, ,where F_o and F_c are the observed and calculated structure factor amplitudes , respectively.

[d] The R_{free} was calculated with 5% of the data excluded from the refinement.

Supplementary Table S2. Experimental condition of X-ray diffraction.

	γ-M	α-M	**2**	γ-M - Br	α-M - Br
X-ray source	PF-AR	PF-AR	PF-AR	PF-AR	PF-AR
beamline	NE-3A	NW12	NW12	NE-3A	NE-3A
Detector	ADSC Q270	ADSC Q210	ADSC Q210	ADSC Q270	ADSC Q270
Exposure time (s)	0.2	1.8	6.0	0.3	0.4
Detector distance (mm)	167.9	117.3	166.2	188.6	188.6
Oscillation width (°)	0.4	1.0	1.0	1.0	1.0
Frame number	500	180	300	300	300
Oscillation range (°)	200	180	300	300	300

Supplementary Figure S1. The EC_{50} values and *t*-test.

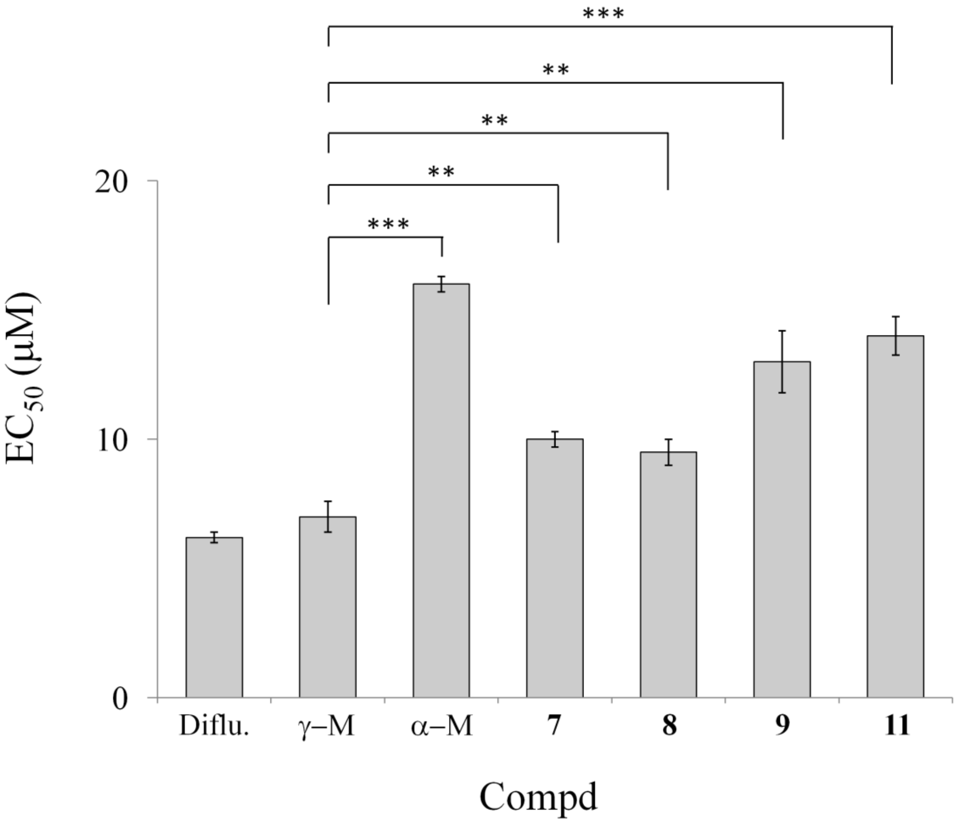

Supplementary Figure S1. One-tailed *t*-test was performed between γ-M and the selected compounds with 0.05 of significance level. ** indicates $p < 0.01$ and *** indicates $p < 0.001$.

Supplementary Figure S2. Concentration-dependent effect in the intrinsic fluorescent and resveratrol competitive experiments.

(a) (b)

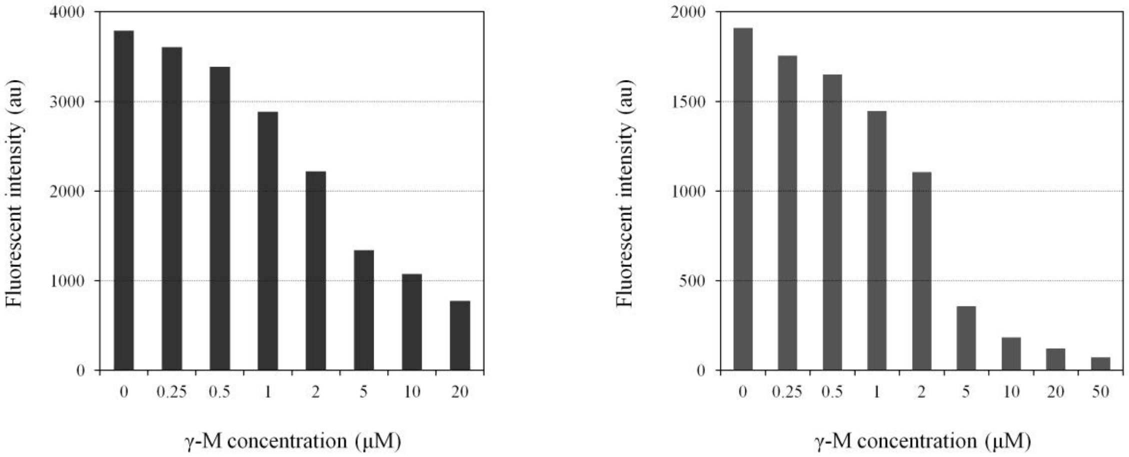

Supplementary Figure S2. (a) Fluorescent intensities at 340 nm at the various γ–M concentrations in the intrinsic fluorescent experiments. (b) Fluorescent intensities at 400 nm at the various γ-M concentrations in the resveratrol competitive binding assay.

Supplementary Figure S3. The quaternary structural stability of V30M mutant TTR in the presence of compounds.

(a)

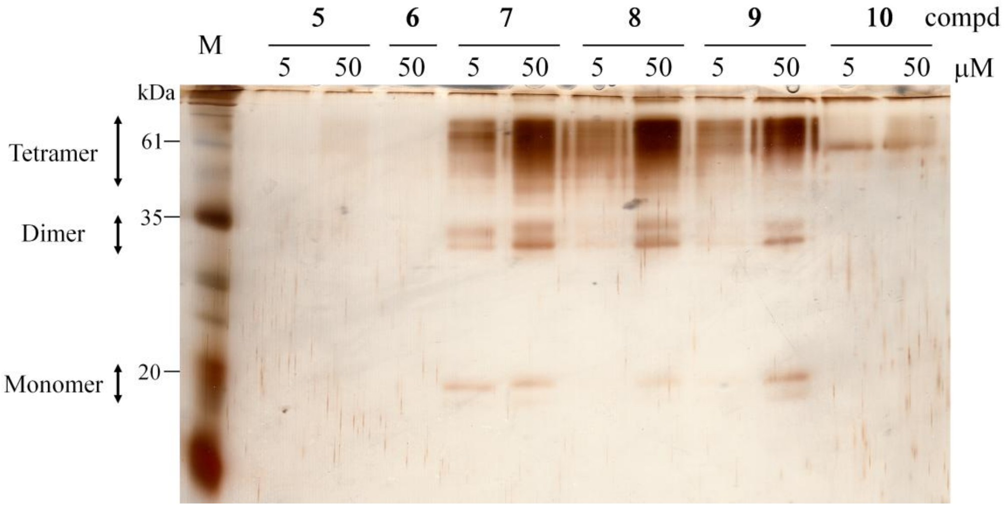

(b)

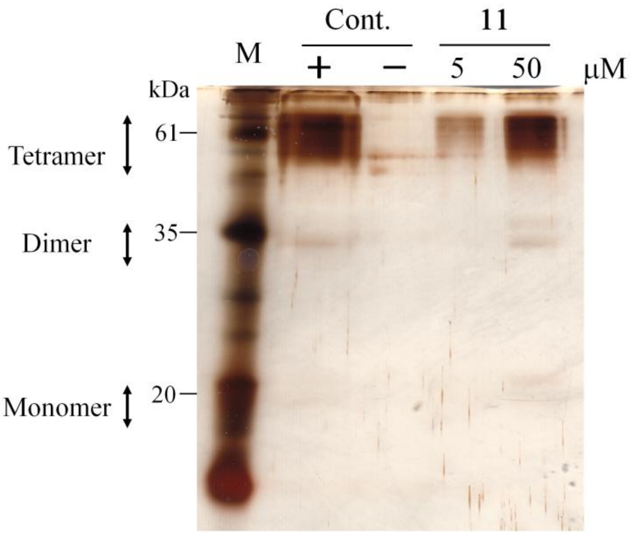

Supplementary Figure S3. Glutaraldehyde cross-linking experiments using compounds **5**, **6**, **7**, **8**, **9** and **10** (a) and the control and **11** (b). The concentrations of the compounds were 5 and 50 µM. The + lane of Cont. indicates the positive control incubated at pH 8.0 without the compounds. The – lane indicates the negative control incubated at pH 4.5 without the compounds.

Supplementary Figure S4. The relative tetramer fractions at 50 μM compounds and inhibition ratio at 20 μM compounds.

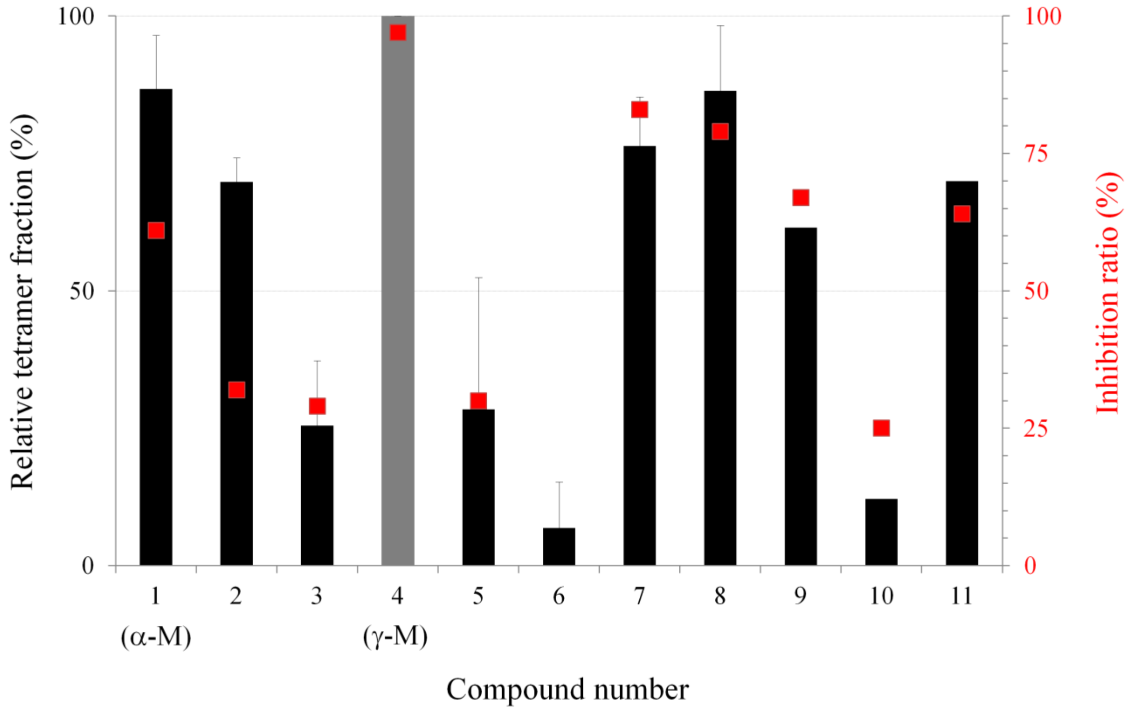

Supplementary Figure S4. The tetramer fractions at 50 μM compounds relative to γ–M (3.6 μM V30M) were indicated as the column bars with the standard deviations (left side axis). The tetramer fractions were quantified by the glutaraldehyde cross-linking experiments. Inhibition ratios were indicated as red-filled square (right side axis). The The inhibition ratio of **6** was not determined because of its low inhibitory potency.

Supplementary Figure S5. Resveratrol competitive assay including the spectra of free γ-M and Resveratrol.

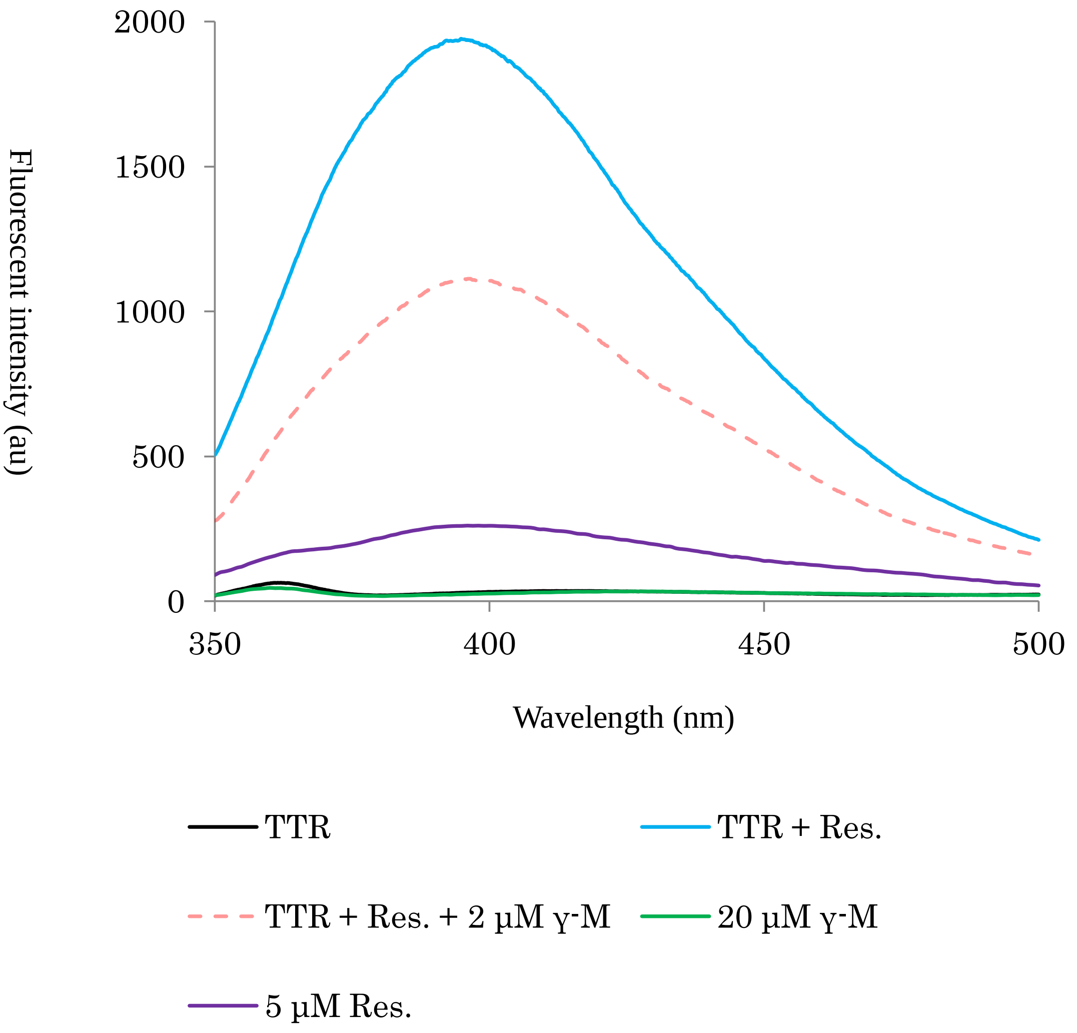

Supplementary Figure S5. The fluorescence spectra (350–500 nm) of resveratrol (5 μM) bound to V30M and on addition of γ-M by excitation at 320 nm.
